# Fomites Could Determine Severity of SARS-CoV-2 Outbreaks in Low-Density White-Tailed Deer (*Odocoileus virginianus*) Populations

**DOI:** 10.1155/tbed/1352911

**Published:** 2025-06-04

**Authors:** Elias G. Rosenblatt, Jonathan D. Cook, Graziella V. DiRenzo, Evan H. Campbell Grant, Michael C. Runge, Brittany A. Mosher

**Affiliations:** ^1^Rubenstein School of Environment and Natural Resources, University of Vermont, Burlington, Vermont, USA; ^2^Eastern Ecological Science Center, U.S. Geological Survey, Laurel, Maryland, USA; ^3^Massachusetts Cooperative Fish and Wildlife Research Unit, U.S. Geological Survey, University of Massachusetts, Amherst, Massachusetts, USA; ^4^Department of Environmental Conservation, University of Massachusetts, Amherst, Massachusetts, USA; ^5^S.O. Conte Anadromous Fish Research Laboratory, Eastern Ecological Science Center, U.S. Geological Survey, Turners Falls, Massachusetts, USA

**Keywords:** fomite, indirect transmission, SARS-CoV-2, transmission risk, white-tailed deer

## Abstract

The establishment of a reservoir species for zoonotic diseases is concerning for both animal and human health. Severe acute respiratory syndrome coronavirus (SARS-CoV)-2, the coronavirus responsible for the COVID-19 pandemic, has been detected in white-tailed deer (*Odocoileus virginianus*) in the United States. Since its initial detection, various studies have documented circulation and evolution of SARS-CoV-2 in deer, with human cases suspected of spill-back from infectious deer. A priority for mitigating SARS-CoV-2 outbreaks in deer populations is determining the contribution of direct (via aerosols and physical contact) and indirect (via contaminated objects and media) transmission pathways. We expanded existing epidemiological models founded on direct transmission pathways to include three indirect transmission pathways of infection for simulated deer populations, including contaminated water, food waste, and feed piles. Despite lower infection probabilities and transmission hazards (measured by force-of-infection (FOI)) posed solely by these indirect pathways compared to direct transmission pathways, the addition of indirect transmission pathways increased FOI, which had ramifications for the severity of SARS-CoV-2 outbreaks in simulated deer populations, particularly in populations with low degrees of spread between deer (measured by basic reproductive number; *R*_0_). We used contact rate models to estimate SARS-CoV-2 spread across deer range in the United States and identified widespread potential for indirect transmission to increase the severity of outbreaks in low-density deer populations. These results indicate that indirect transmission pathways need to be considered in the management of white-tailed deer as a reservoir species for SARS-CoV-2.

## 1. Introduction

Zoonotic diseases, or those that are transmitted between human and nonhuman hosts, account for over 60% of emerging infectious diseases in humans [1]. The maintenance of zoonotic pathogens in animal hosts is a threat to animal and human health, as these reservoirs increase the potential for pathogens to evolve or recombine into new strains or variants that have different epidemiological and pathological characteristics [2]. The persistence of pathogens in reservoir hosts can have broader population and ecosystem effects, in addition to risks to human public health [3]. Reservoir hosts can transmit pathogens to other susceptible species, leading to disease that can negatively impact host populations [4] and disrupt trophic interactions in the broader ecosystem [5]. Preventing the establishment of zoonotic disease reservoirs in nonhuman hosts is a priority for managing threats to public health [2, 6]. A key to prevention is a better understanding of transmission pathways that are critical to the introduction and persistence of zoonotic disease in potential reservoir species.

Coronaviruses are viral pathogens with broad host ranges and are responsible for numerous epizootics [7]. Coronaviruses have rapid mutation rates, which can lead to increased zoonotic potential during prolonged circulation in nonhuman hosts [8]. While zoonotic coronaviruses have emerged from bat species, multiple other animal species have served as intermediate or reservoir hosts, including masked palm civets (*Paguma larvata*) for severe acute respiratory syndrome coronavirus (SARS-CoV)-1 and dromedary camels (*Camelus dromedarius*) for Middle East respiratory syndrome coronavirus (MERS-CoV) [9–11]. The global pandemic caused by SARS-CoV-2 may have originated in horseshoe bats (*Rinolophus spp*.) and subsequently spread to other wildlife species, posing risks of nonhuman reservoirs producing emergent variants that could reinfect humans [8, 12].

One SARS-CoV-2 host of concern in the United States is white-tailed deer (*Odocoileus virginianus*, hereafter deer) because of its susceptibility, sociality with conspecifics, broadscale distribution, high abundance in some habitats, and potential for frequent interactions with humans. The captive deer industry, with 200,000 deer in captivity in 2012, adds an additional component of disease spillover potential to wild deer via fenceline transmission from captive to wild deer [13]. This concern is underscored by repeated detections of SARS-CoV-2 in captive and wild deer populations across broad geographical ranges [14]. Moreover, some SARS-CoV-2 variants circulating in deer populations descend from earlier human variants of concern that, once introduced into deer, have continued to adapt at a faster rate than observed in humans [15, 16]. These divergent variants have the potential to spillback to humans and affect the severity of disease and efficacy of treatments [15, 17].

**Figure 1:**
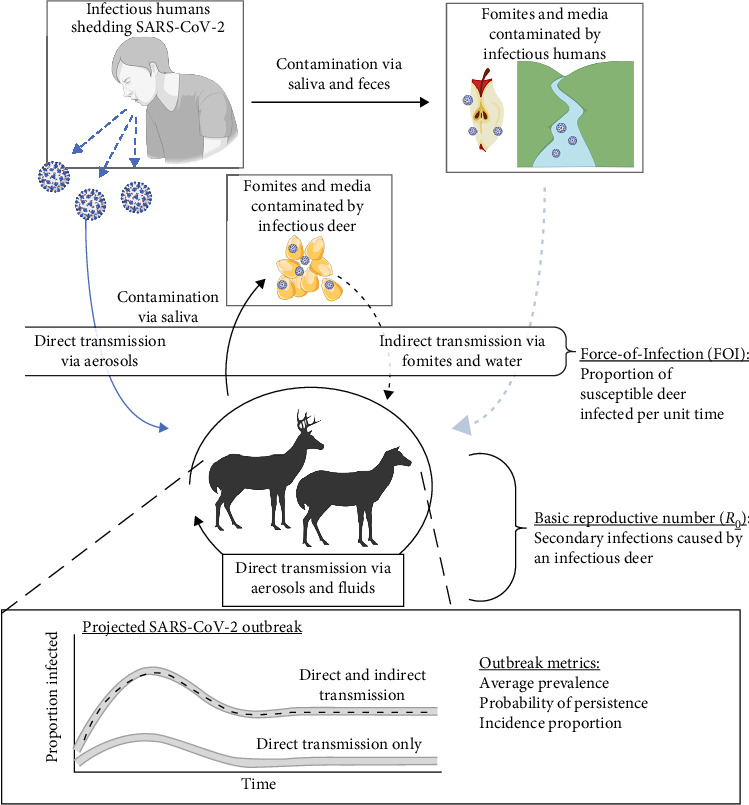
A conceptual diagram of the epidemiological effects of indirect transmission in addition to direct transmission in SARS-CoV-2 spillover and spread in white-tailed deer (*Odocoileus virginianus*). Spillover occurs when infectious humans either infect deer through direct transmission via aerosols (solid blue arrow) or indirectly through contaminated fomites and media (dashed blue line). Once deer are infected, SARS-CoV-2 outbreaks are maintained by direct transmission between deer (solid black arrow) or through contaminated fomites and media (dashed black arrow). We quantified the per-capita rate that deer become infected (FOI) and the spread of SARS-CoV-2 within a deer population (basic reproductive numbers; *R*_0_). We projected SARS-CoV-2 prevalence in a deer population and related increases in FOI in two scenarios, one with direct and indirect transmission pathways and one with only direct transmission and we quantified how each transmission scenario contributed to changes in outbreak metrics. Created in BioRender. Rosenblatt, E. (2025; https://BioRender.com/g60d995).

Decision makers need accurate predictions of SARS-CoV-2 outbreak dynamics to better inform disease mitigation across wildlife, agricultural, and human health sectors (collectively termed One Health). There is a lack of information regarding the contributions of direct and indirect transmission pathways that lead to the persistence of SARS-CoV-2 in deer (Figure 1). The tissue tropism of SARS-CoV-2 favors upper respiratory tract tissues in humans, deer, and other wildlife species [18]. However, broader tissue tropism of SARS-CoV-2 in the oral cavity and nasopharynx suggests that indirect transmission through ingestion and contact with contaminated fomites and media poses another potential, but understudied, contribution to SARS-CoV-2 infection in deer populations, as observed in other wildlife diseases [19–21]. As deer continue to be infected with SARS-CoV-2 across the United States [22], ignoring the contributions of indirect transmission pathways may lead to incorrect assessments of population vulnerability to continued circulation of SARS-CoV-2 and the role of deer as a reservoir species. In total, this incomplete picture may limit management and regulatory opportunities across One Health sectors to reduce spread and evolution of novel variants in deer.

We evaluated direct and indirect pathways for their ability to sustain disease transmission in simulated wild deer populations (Figure 1). We modified existing epidemiological models of direct SARS-CoV-2 transmission in deer and humans to incorporate indirect transmission from three pathways, including ingestion of contaminated water sources, human food waste, and supplemental feed. We identify the epidemiological conditions under which outbreak severity is most sensitive to the cumulative risks posed by direct and indirect transmission and describe the spatial distribution of hazards according to varying conditions across deer range. Our simulation results highlight the importance of considering a broader range of transmission pathways when evaluating the potential for pathogen reservoirs to maintain infections in animal species and identify potential pathways that drive long-term spread and the evolution of novel variants which may be targeted for management.

## 2. Materials and Methods

We use three analyses to evaluate the contribution of indirect transmission to SARS-CoV-2 outbreaks in deer. First, we estimated the probability of infection and force-of-infection (FOI) for susceptible deer from three indirect transmission pathways: deer drinking from contaminated water sources, deer consuming contaminated food waste, and deer eating at communal and supplemental feed piles. We compared probability of infection and FOI for direct transmission from infectious humans via aerosols using estimates from Rosenblatt et al. [23]. We characterized the increase of cumulative FOI when considering both direct and indirect transmission pathways. Second, we extended the susceptible-infectious-recovered-susceptible (SIRS) approach used in [23] to project SARS-CoV-2 outbreaks in wild deer given both direct and indirect transmission pathways. We used these results to understand the sensitivity of various outbreak metrics to changes in FOI and spread of SARS-CoV-2 between deer (basic reproductive number; *R*_0_). Finally, with these insights into outbreak sensitivity to FOI and *R*_0_, we identified portions of the deer range in the contiguous United States where the additional hazards of indirect transmission pathways could alter SARS-CoV-2 outbreaks.

We focused on quantifying the contribution of indirect transmission in the introduction and spread of SARS-CoV-2 in wild deer inhabiting suburban areas (100 humans/km^2^) [24], because the indirect transmission pathways outlined here are clearly contextualized in a suburban setting. Calculations were conducted in R [25] and parameter values used in these calculations are listed in Table S1. The R code and associated data are publicly available from Rosenblatt et al. [26].

### 2.1. Probability of Infection

We used parameters from published studies to estimate the dose received by a susceptible deer through each indirect transmission pathway (Table S1). We simulated 1000 exposure events for each pathway, to capture the uncertainty in the underlying parameters. With these resulting dosages, we used a Wells–Riley dose–response model to estimate the probability of infection (*σ*_pathway_) given the dose received as:

(1) $$\begin{array}{c} \sigma_{\text{pathway}}=1-{\text{e}}^{-\frac{{\text{Dose}}_{\text{pathway}}\times p_{\text{ingested}}}{K}}, \end{array}$$

where *K* is the reciprocal of the probability of a single plaque-forming unit (PFU) causing infection and *p*_ingested_ is the proportional reduction of dose received, Dose_pathway_, via ingestion relative to inhalation.

Contaminated water sources: We calculated probability of SARS-CoV-2 infection from human wastewater to a wild deer drinking from a contaminated water source. To simplify our calculations and to be conservative in our estimates, we assumed that contaminated wastewater was instantly well-mixed into natural waterways and that the virus did not settle into the sediment before ingestion by the deer.

We began with a predicted viral load of 1.09 PFU/ml_raw sewage_ (standard error = 0.04), based on a linear relationship between SARS-CoV-2 positivity and the concentration of PFUs (PFU/ml_raw sewage_) in contaminated sewage [27] and 5% prevalence in a human population (Table S1). We modeled the reduction in SARS-CoV-2 concentration in sewage as it passed through a centralized treatment facility. These facilities serve 82% of households in the United States [28] and treat 93.1% of the wastewater they receive [29]. Assuming wastewater is successfully processed by a treatment facility, we applied randomly drawn log reductions (LRs) in SARS-CoV-2 concentration from a uniform distribution (3–5 LR), ranging from primary and conventional activated sludge secondary treatments to more intensive tertiary treatments [27, 30, 31]. We calculated the post-treatment concentration of SARS-CoV-2 virions (PFU/ml) in contaminated water as:

(2) $$\begin{array}{c} \text{PFU}/{\text{ml}}_{\text{posttreatment},t=0}=\frac{\text{PFU}/{\text{ml}}_{\text{raw sewage}}}{10^{\text{LR}}}. \end{array}$$

After wastewater treatment, treated wastewater is discharged and diluted into natural water bodies where virions decay. We modeled this process as:

(3) $$\begin{array}{c} \text{PFU}/{\text{ml}}_{\text{final}}=\text{PFU}/{\text{ml}}_{\text{posttreatment},t=0}\times p_{\text{dilution}}\times e^{-\rho_{\text{decay},\text{water}}\times t_{\text{decay}}}. \end{array}$$

We calculated dilution at discharge (*p*_dilution_) by randomly sampling wastewater ratios for all river reaches overlapping with deer range in the contiguous United States (Table S1) [32, 33]. We excluded reaches with no wastewater effluent contribution and reaches with ratios above the 99.5%, presuming the remaining 0.5% of reaches are in settings that would discourage the use by deer. We considered various residency times in waterways ranging from 0 to 96 h (*t*_decay_) and applied a viral decay rate (*ρ*_decay, water_). We then calculated the dose received by a deer ingesting its entire daily water requirement from contaminated water sources (DWR; Equation 4).

(4) $$\begin{array}{c} {\text{Dose}}_{\text{wastewater}}=\text{PFU}/{\text{ml}}_{\text{final}}\times \text{DWR}. \end{array}$$

Contaminated food waste: We calculated the probability of SARS-CoV-2 infection for a susceptible deer eating a single discarded apple core that was contaminated by an infectious human.

We calculate the initial viral load on the discarded apple core (PFU_apple core,⁣*t*=0_) as:

(5) $$\begin{array}{c} {\text{PFU}}_{\text{apple core},t=0}=\frac{{\text{gc}}_{\text{SARS-CoV-2}}/{\text{ml}}_{\text{human saliva}}}{{\text{gc}}_{\text{SARS}-\text{CoV}-2}/{\text{PFU}}_{\text{SARS-CoV-2}}}\times \left(2\pi r_{\text{apple core}}h_{\text{apple core}}\right)\times d_{\text{saliva}}\times p_{\text{saliva transfer}}. \end{array}$$

We converted published human salivary viral load ($\frac{\text{genomic copies }{\left[\text{gc}\right]}_{\text{SARS}-\text{CoV}-2}}{{\text{ml}}_{\text{human saliva}}}$) to PFU concentration using an estimate of the genomic copies to PFU, gc_SARS−CoV−2_/PFU_SARS−CoV−2_. We calculated the volume of human saliva deposited on the apple core as the product of the surface area of the apple core, approximating the apple core as a cylinder with radius *r*_apple core_ and height *h*_apple core_, the depth of saliva in the human's mouth (*d*_saliva_), and the proportion of that saliva transferred to the apple core (*p*_saliva transfer_). We estimated a range of apple core sizes by measuring a small and large apple. The core radii were 1.0 and 1.6 cm and heights were 5.4 and 7.6 cm, respectively.

We calculated the remaining viral load on the apple core (PFU_apple core,*t*_) and the dose (Dose_foodwaste_) received by the susceptible deer after decay (*ρ*_decay,fomite_) over a range of time (1 min–96 h; *t*_decay_), using the exponential decay function:

(6) $$\begin{array}{c} {\text{Dose}}_{\text{foodwaste}}={\text{PFU}}_{\text{apple core},t}={\text{PFU}}_{\text{apple core},t=0}\times e^{-\rho_{\text{decay},\text{fomite}}\times t_{\text{decay}}}. \end{array}$$

We assumed that the apple core was completely consumed by a susceptible deer.

Contaminated feed pile: We calculated the probability of SARS-CoV-2 infection of a susceptible deer eating from a supplemental feed pile that was previously contaminated by another infected deer. This contamination process begins with the surface area contacted by the saliva of an infected deer, as:

(7) $$\begin{array}{c} A_{\text{contacted}}=\sum_{1}^{c}p_{{\text{contacted}}_{c}}\times {\text{SA}}_{c}. \end{array}$$

We assumed that an infectious deer came to feed and while feeding it touched and contaminated anywhere from 1 to 1000 corn kernels (c). We randomly assigned each kernel a proportion of its surface area (SA_*c*_) that was contaminated with saliva from the infectious deer, ranging from 0.01 to 0.5 (*p*_contacted_*c*__). Using the sum of these surface areas across all kernels contacted (*A*_contacted_) and the same saliva thickness (*d*_saliva_) and proportion of saliva transferred (*p*_saliva transfer_) used previously in the contaminated food waste scenario (Equation 5), we calculated the initial viral load on contaminated kernels as:

(8) $$\begin{array}{c} {\text{PFU}}_{\text{feed pile},t=0}=\frac{\frac{{\text{gc}}_{\text{SARS-CoV-2}}}{{\text{ml}}_{\text{deer saliva}}}}{{\text{gc}}_{\text{SARS-CoV-2}}/{\text{PFU}}_{\text{SARS-CoV-2}}}\times A_{\text{kernelcontacted}}\times d_{\text{saliva}}\times p_{\text{saliva transfer}}. \end{array}$$

We assumed the viral load in the feed pile goes through the same decay process as detailed in the contaminated food waste scenario (Equation 9). We assume the same saliva thickness and range of saliva transfer for deer (Equation 8) and humans (Equation 5). We assumed that a susceptible deer consumes all the contaminated kernels and calculated the dose received (PFU_feed pile,*t*_). We limited maximum possible number of contaminated kernels consumed to 1000, averaging 245 g [34]. This serving size is conservative relative to published supplemental feed studies (e.g., 400 g shelled corn per deer, per day) [35].

(9) $$\begin{array}{c} {\text{Dose}}_{\text{feed pile}}={\text{PFU}}_{\text{feed pile},t}={\text{PFU}}_{\text{feed pile},t=0}\times e^{-\rho_{\text{decay},\text{fomite}}\times t_{\text{decay}}}. \end{array}$$

### 2.2. FOI

We converted the probability of SARS-CoV-2 infection given exposure for each indirect pathway to transmission risk (*β*_indirect_^pathway^) as:

(10) $$\begin{array}{c} \beta_{\text{indirect}}^{\text{pathway}}=\sigma_{\text{pathway}}\times c_{\text{pathway}}, \end{array}$$

or the product of the probability of infection given exposure to an indirect pathway (*σ*_pathway_) and the exposure rate to that pathway (*c*_pathway_). We calculate the FOI (FOI_pathway_), which is the per-capita rate that deer become infected, as the product of transmission risk (*β*_indirect_^pathway^) and the prevalence in the host species *i* (human or deer) that contaminates the pathway, or the prevalence of contaminated surfaces (*i*_*i*,pathway_), that is,

(11) $$\begin{array}{c} {\text{FOI}}_{\text{pathway}}=\beta_{\text{indirect}}^{\text{pathway}}\times i_{i,\text{pathway}}. \end{array}$$

We calculated the probability of infection (*σ*_pathway_) for contaminated water sources and fomites above (Equation 1). We simulated 1000 iterations of exposure rates to pair with the 1000 iterations of infection probability calculations detailed above. We compared resulting FOI estimates across pathways and with FOI estimates for direct transmission via aerosols from humans [23].

Contaminated water sources: We assumed that a susceptible deer ingests 100% of its daily water requirement from waterbodies that could be contaminated with wastewater (*c*_water_ = 1). Transmission risk from contaminated water sources was, therefore, equal to the calculated infection probability above, or

(12) $$\begin{array}{c} \beta_{\text{indirect}}^{\text{water}}=\sigma_{\text{water}}\times c_{\text{water}}. \end{array}$$

The FOI for contaminated water sources (FOI_water_) was calculated by multiplying *β*_*indirect*_^*water*^ by the prevalence rate (*i*_water_) in waterbodies, or

(13) $$\begin{array}{c} {\text{FOI}}_{\text{water}}=\beta_{\text{indirect}}^{\text{water}}\times i_{\text{water}.} \end{array}$$

The prevalence rate in waterbodies is the product of the proportion of surface water in rivers and streams (*p*_rivers_) and the proportion of those rivers and streams contaminated by wastewater effluent (*p*_contaminated_), or,

(14) $$\begin{array}{c} i_{\text{water}}=p_{\text{rivers}}\times p_{\text{contaminated}}. \end{array}$$

We calculated *p*_rivers_ and *p*_contaminated_ for each county across the deer range in the contiguous United States (Table S1). The county-level *i*_water_ estimates were used to generate 1000 random draws to pair with our 1000 random draws of *β*_indirect_^water^, to calculate FOI_water_.

Contaminated food waste: We assumed that a susceptible deer ingests 0–10 contaminated apple cores per day, drawn from a uniform distribution (*c*_food waste_). We have no empirical estimates available for this consumption rate, so we set this upper bound to represent exposure from backyard compost piles. Prevalence in humans (*i*_*H*_) was fixed at 5% to match conditions described in Rosenblatt et al.'s [23] epidemiological models.

Contaminated feed pile: We assumed that a susceptible deer ingests zero to one servings of feed contaminated by infectious deer, drawn from a uniform distribution, and assumed that this exposure rate is frequency dependent. Prevalence in wild deer (*i*_*W*_), the source of contamination in food waste, was drawn from daily prevalence values in Rosenblatt et al.'s [23] epidemiological models (mean = 2.4%; range: 0%–95.1%). We calculated the cumulative FOI (FOI_cumulative_) across both direct and indirect pathways for spillover from humans (Equation 15) and compared this to FOI for direct transmission from humans via aerosols (FOI_HW_^Aero^) [23].

(15) $$\begin{array}{c} {\text{FOI}}_{\text{cumulative}}={\text{FOI}}_{\text{HW}}^{\text{Aero}}+{\text{FOI}}_{\text{indirect}}^{\text{food waste}}+{\text{FOI}}_{\text{indirect}}^{\text{water}}. \end{array}$$

We did not include feedpile transmission in this calculation as we were interested in the additional spillover hazard posed by human-contaminated fomites and water. We converted our continuous metric of direct transmission FOI to four categories using the getJenksBreaks() function from the BAMMtools package [36] to visualize sensitivity patterns. We also assigned cumulative FOI values with both direct and indirect transmission to these categories. We summarized the proportion of simulations that increased in FOI category with the inclusion of indirect transmission pathways.

### 2.3. Epidemiological Modeling

We used SIRS ordinary differential equations (ODEs) to project proportions of the wild population that are susceptible (*s*_*W*_), infected (*i*_*W*_), and recovered (*r*_*W*_), starting with a naïve population (*s*_*W*,*t*=0_ = 1;  *i*_*W*,*t*=0_ = *r*_*W*,*t*=0_ = 0). We projected continuous SARS-CoV-2 introduction and spread over a 120-day fall season, considering both direct and indirect transmission pathways (Table S2). We ran 1000 iterations of a simulated outbreak for a wild deer population, with and without the presence of intensive captive facilities that would allow fenceline transmission between captive and wild deer (referred to as “wild-captive complex” and “wild,” respectively). Each iteration had a randomly drawn parameter set. We projected the proportion of deer that were susceptible, infectious, or recovered for 120 days for each iteration, using the ODE solver *ode*() from the deSolve package in R [25, 37]. We excluded feedpile transmission from the SIRS approach because our focus was to examine the role of indirect transmission from human-contaminated water sources and fomites.

We characterized outbreak dynamics for the SIRS ODE set using three metrics. First, we estimated the average prevalence of deer ($\overset{―}{i_{W}}$) across the 120-day projection. Second, we used the *runsteady*() function from the rootSolve package [38, 39] to determine if SARS-CoV-2 would persist beyond the 120-day projection, with a threshold of at least one deer infected out of 1000 (>0.1%). We estimated mean probability of persistence and 95% binomial confidence intervals using the *binom.confint*() function with the exact method from the binom package [40]. Finally, we tracked the incidence proportion, or cumulative proportion of the population infected over the 120 days, as:

(16) $$\begin{array}{c} \text{Incidence proportion, wild}=\sum_{t=1}^{120}s_{W,t-1}\times \left(\beta_{\text{WW}}^{\text{Aero}}i_{W,t-1}+\beta_{\text{WW}}^{\text{DC}}i_{W,t-1}+\beta_{\text{HW}}^{\text{Aero}}i_{H}+\beta_{\text{Indirect}}^{\text{Water}}i_{\text{Water}}+\beta_{\text{Indirect}}^{\text{Food waste}}i_{H}+\beta_{\text{Indirect}}^{\text{Feed pile}}i_{W,t-1}\right). \end{array}$$

or

(17) $$\begin{array}{c} \text{Incidence proportion, wild-captive complex}=\sum_{t=1}^{120}s_{W,t-1}\times \left(\beta_{\text{WW}}^{\text{Aero}}i_{W,t-1}+\beta_{\text{WW}}^{\text{DC}}i_{W,t-1}+\beta_{\text{CW}}^{\text{Aero}}i_{C,t-1}+\beta_{\text{CW}}^{\text{DC}}i_{C,t-1}+\beta_{\text{HW}}^{\text{Aero}}i_{H}+\beta_{\text{Indirect}}^{\text{Water}}i_{\text{Water}}+\beta_{\text{Indirect}}^{\text{Food waste}}i_{H}+\beta_{\text{Indirect}}^{\text{Feed pile}}i_{W,t-1}\right). \end{array}$$

We hypothesized that the sensitivity of a SARS-CoV-2 outbreak to the inclusion of indirect transmission may differ based on *R*_0_ or the number of additional deer infected by an infectious deer and FOI. We calculated *R*_0_ for all combinations of transmission risk and contexts based on direct transmission rates via aerosols (*β*_WW_^Aero^) and fluid transferred on contact (*β*_WW_^DC^), divided by the duration of recovery from SARS-CoV-2 infection in deer (*γ*), as:

(18) $$\begin{array}{c} R_{0}=\frac{\beta_{\text{WW}}^{\text{Aero}}+\beta_{\text{WW}}^{\text{DC}}}{\gamma }. \end{array}$$

We binned outbreak metrics from each simulation by *R*_0_ and FOI categories to test the sensitivity of outbreaks to the addition of indirect transmission. We defined *R*_0_ categories of *R*_0_ < 0.5, 0.5 ≤ *R*_0_ ≤ 1, 1 < *R*_0_ ≤ 2, 2 < *R*_0_ ≤ 3, and 3 < *R*_0_, spanning conditions where spread is unlikely, spread is characterized by stuttering chains, spread is likely sustained, and disease is widespread, respectively [41]. We summarized median outbreak metrics and 95% confidence for each combination of *R*_0_ and FOI category. We report this summary for wild deer simulations in the main text and report results for the wild–captive complex simulations in the supplemental materials.

### 2.4. Areas of Wild Deer Range Sensitive to Increases in FOI

We used the proximity rate model developed by Habib et al. [42] to estimate deer–deer contact rates and median *R*_0_ across gradients of wooded habitat availability and deer density. This modeling approach described wooded habitat availability [43] and deer density [44] for 57% of the deer range containing lower density populations in the contiguous United States. Based on our sensitivity results, we identified combinations of wooded habitat availability and deer density that would predict *R*_0_ values sensitive to the additional hazard posed by indirect transmission pathways. We estimated contact rates and median *R*_0_ values across the deer range in the contiguous United States using land cover [43] and deer density [44] geospatial datasets rescaled to 100 km^2^ pixel resolution in QGIS (version 3.32.3-Lima; qgis.org). We note that the range-wide deer density dataset used for this analysis [44] presents data from 2003. To our knowledge, there are no equivalent datasets available from a more recent time period. We reported the proportion of pixels in each state that had any probability of median *R*_0_ falling within ranges sensitive to additional FOI from indirect transmission.

## 3. Results

**Figure 2:**
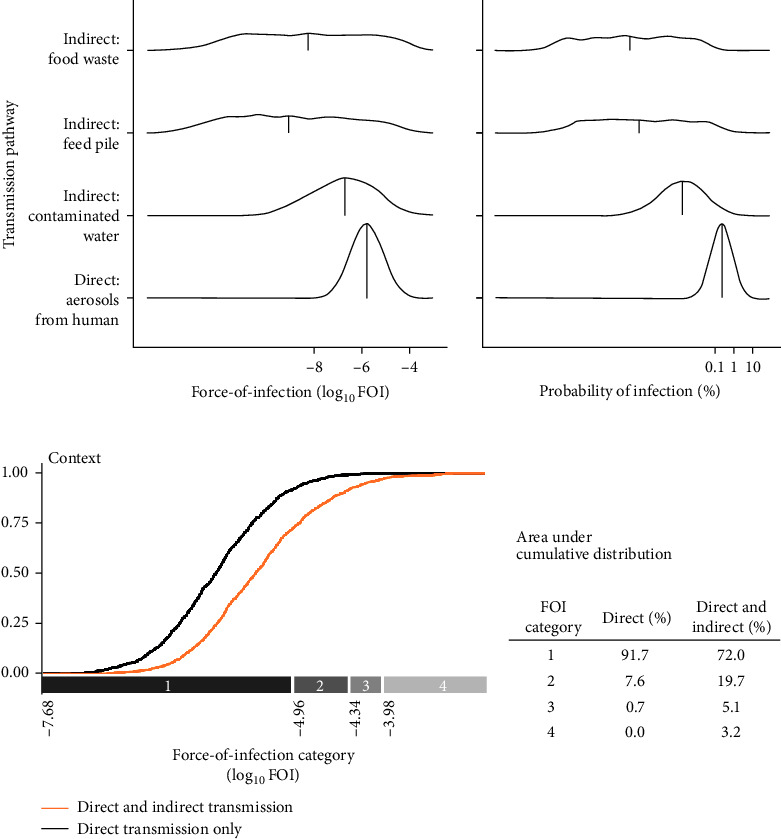
SARS-CoV-2 infection characteristics of indirect and direct transmission pathways of spillover to white-tailed deer (*Odocoileus virginianus*) (A). Distributions of FOI and probability of infection given exposure are summarized by quartiles along a log_10_-transformed axis. Cumulative FOI increased when both direct and indirect transmission are considered (B). Shifts in cumulative probability distributions for four FOI categories are summarized as the proportion of simulations falling within each category. Break points for FOI categories are indicated on the *x*-axis (log_10_-scale).

Indirect transmission pathways were an important driver of SARS-CoV-2 dynamics in simulated wild deer populations because they increased the overall hazard of SARS-CoV-2 transmission (FOI) from humans to wild deer and the maintenance of simulated outbreaks. Relative to direct transmission from humans to deer via aerosols, indirect transmission pathways had lower and more variable FOI and were less likely to lead to human-to-deer infection (Figure 2A). Median FOI for pathways associated with contaminated water sources, human food waste, and deer feed piles were 10.5%, 0.5%, and <0.1% of the median FOI for aerosolized transmission from humans, respectively. The median probability of SARS-CoV-2 infection for these indirect pathways were all <0.1% of the median probability of infection for aerosolized transmission from humans. Despite small relative values, spillover hazards from indirect pathways, when added to hazards from direct transmission, resulted in an overall increase in FOI (Figure 2B).

### 3.1. Indirect Transmission Pathways Pose Additional, Highly Variable Hazards of Spillover of SARS-CoV-2 to Wild Deer Populations

When we categorized FOI values by their natural breaks, we estimated that the addition of indirect transmission pathways to aerosol transmission from humans increased the number of simulations with higher FOI categories (categories 2–4) from 8.3% to 28% (Figure 2B). FOI rates increased by a median of 38.5% across all simulations with the addition of indirect transmission pathways, with high variation among simulations (2.5% quantile = 0.03%, 97.5% quantile = 14,633%). Most of these increases in cumulative FOI resulted in shifts from the lowest FOI category to the second lowest FOI category (70.4%), yet a substantial proportion (29.6%) of these cumulative FOI increases resulted in shifts to higher FOI categories (categories 3 and 4; Figure 2B).

### 3.2. When R_0_ is Low, Indirect Transmission Pathways Could Increase the Severity of a SARS-CoV-2 Outbreak in Wild Deer

**Figure 3:**
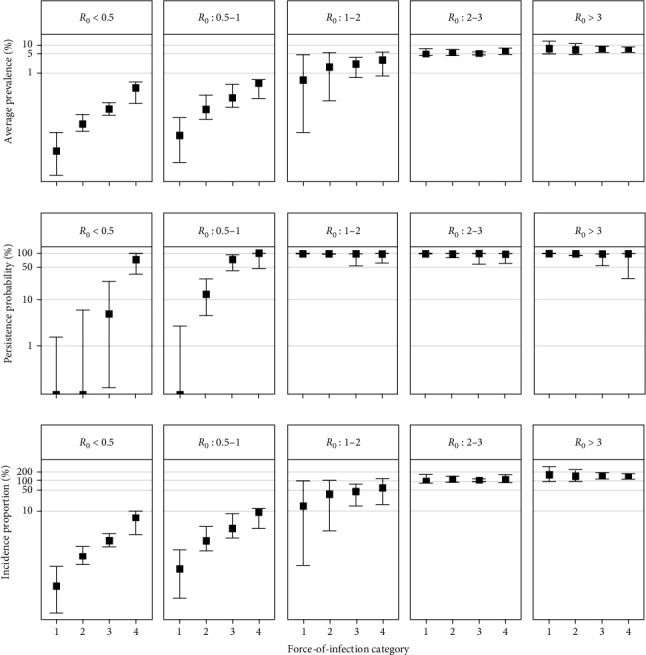
Sensitivities of average prevalence, persistence probability, and incidence proportion to categories of spillover (FOI) and spread amongst white-tailed deer (*Odocoileus virginianus*; *R*_0_) in a wild population exposed to both direct and indirect transmission pathways. We defined *R*_0_ categories of *R*_0_ < 0.5, 0.5 ≤ *R*_0_ ≤ 1, 1 < *R*_0_ ≤ 2, 2 < *R*_0_ ≤ 3, and 3 < *R*_0_, spanning conditions where spread is unlikely, spread is characterized by stuttering chains, spread is likely sustained, and spread is widespread, respectively [23]. FOI categories include <1.096 × 10^−5^ (1), 1.096 × 10^−5^−4.571 × 10^−5^ (2), 4.571 × 10^−5^−1.047 × 10^−4^ (3), and >1.047 × 10^−4^ (4). Points indicate median values from simulations that fall within each combination of FOI and *R*_0_ categories, with error bars indicating either 2.5 and 97.5 quantiles of simulation values (average prevalence and incidence proportion) or 95% confidence intervals (persistence probability).

With their cumulative FOI hazard, direct and indirect transmission pathways alter disease outbreaks in wild deer compared to outbreaks projected with direct transmission pathways alone. However, the magnitude of this change depended on a simulation's basic reproductive number (*R*_0_) or the number of secondary infections caused by a single infectious deer in a naïve population via direct transmission (e.g., direct contact and aerosol transmission). When we simulated disease outbreaks over a 120-day fall season and partitioned simulations by *R*_0_, we found that average prevalence, persistence probability, and incidence proportion were sensitive to FOI (Figure 3). These sensitivities were especially pronounced in simulations with a resulting *R*_0_ between 0.5 and 1. This *R*_0_ range can lead to stuttering chains of infection that will not be sustained without repeated introduction events, such as those occurring by indirect transmission pathways [41]. We found that in areas with low *R*_0_ values, that the added FOI hazard from indirect transmission pathways could increase the severity of a SARS-CoV-2 outbreak. We did not detect any changes to this sensitivity when wild deer faced additional hazards from fenceline interactions with captive deer also experiencing SARS-CoV-2 outbreaks (Figure S1).

### 3.3. The Potential for Deer to Serve as Reservoir Species for SARS-CoV-2 Is Likely Underestimated

**Figure 4:**
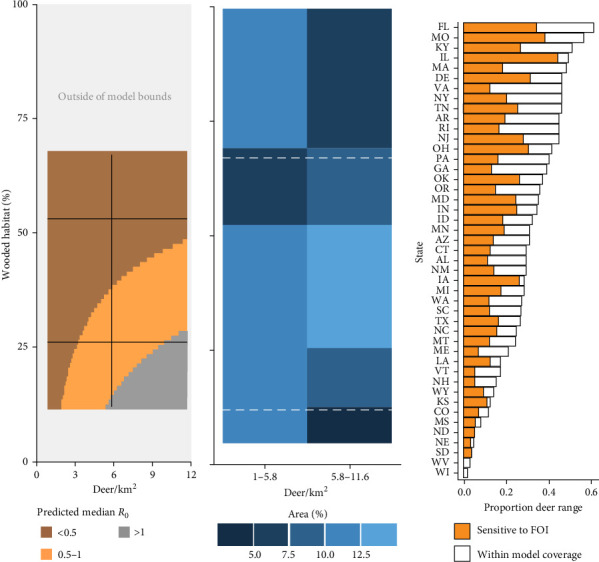
(A) Median transmission of SARS-CoV-2 between white-tailed deer (*Odocoileus virginianus*; *R*_0_) was dependent on deer density and the proportion of wooded habitat available. Using Habib et al.'s [42] contact rate model, we estimated *R*_0_ for areas with 1−11.6 deer/km^2^ and with 12%–63% wooded habitat. The vertical black line indicates the partition between two deer density categories identified by Hanberry and Hanberry's [44] county-level density estimates. Horizontal black lines indicate wooded habitat availability values included in Habib et al.'s [42] contact rate model. We estimated sensitive epidemiological conditions (0.5 < *R*_0_ < 1) across a range of deer density and wooded habitat availability. (B) *R*_0_ estimates covered 57% of low-density white-tailed deer range (between dashed white lines). Note that pixel bounds align with Hanberry and Hanberry's [44] county-level density estimates (vertical bounds) and wooded habitat availability considered by Habib et al. [42] (quantified using 42; horizontal bounds). (C) Forty-five states in the contiguous United States have areas that fall within the scope of inference from Habib et al.'s [42] contact rate model, with variable proportions of deer ranges in these states that are predicted to have epidemiological conditions sensitive to increases in FOI from indirect transmission pathways.

**Figure 5:**
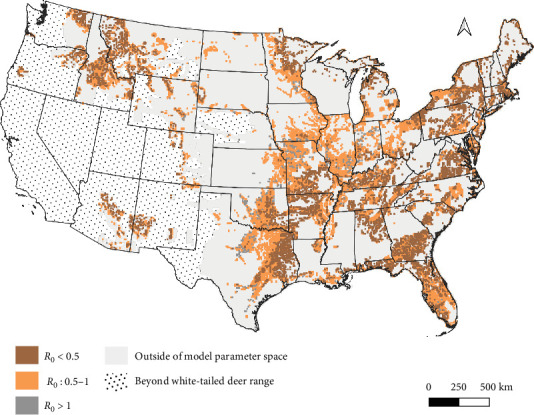
Median spread (*R*_0_) of SARS-CoV-2 between white-tailed deer (*Odocoileus virginianus*) across their range in the contiguous United States. We estimated that outbreaks in areas where *R*_0_ is predicted between 0.5 and 1 have the greatest sensitivity to increases in cumulative FOI, including those increases that may be caused by indirect transmission. Epidemiological models that only consider direct transmission pathways may underestimate the severity of outbreaks in these areas. Predictions were made at a scale of 100 km^2^ pixels across wild deer range, using available county-level deer densities [43] and classifications from the National Land Cover Database classes [42]. Pixels with conditions outside the parameter space of Habib et al.'s [42] contact rate model are indicated by solid light gray coloration.

There is widespread potential that low-density deer populations (≤11.6 deer/km^2^) could be sensitive to increases in spillover hazard from indirect transmission pathways. We mapped combinations of wooded habitat availability and deer density and highlight areas in 45 U.S. states, where we find the combination of deer density and habitat result in expected increases in spillover hazard from indirect transmission (Figures 4 and 5). The proportion of area within the sensitive range of *R*_0_ varied greatly between states, though this was partially influenced by the varying proportion of each state's area that fell within the range of conditions that were supported by our model (Figure 4). Areas with sensitive epidemiological conditions (0.5 ≤ *R*_0_ ≤ 1) were widespread across deer range in the contiguous United States (Figure 5). Our results suggest that the maintenance of SARS-CoV-2 reservoirs in deer is likely to be underestimated if epidemiological models do not consider the contribution of indirect transmission pathways to the spillover hazard.

## 4. Discussion

We found that the severity and persistence of SARS-CoV-2 outbreaks in deer depended, in part, on indirect transmission pathways between humans and wild deer. We estimated lower and more variable probabilities of SARS-CoV-2 infection and FOI for indirect transmission pathways compared to direct transmission from humans through aerosols. However, when we integrated both direct and indirect transmission pathways into our epidemiological models, we found that increases in cumulative FOI was consequential for an outbreak's severity. These differences were especially apparent when deer densities and forested cover favored lower rates of deer contact and deer-to-deer transmission. Recent work suggests that a single SARS-CoV-2 spillover event infecting even a small proportion of a deer populations can result in outbreak dynamics like those resulting from continual direct spillover from humans [23]. Here, we demonstrate that the marginal increase of spillover risk when considering indirect transmission pathways can change the outcome of spillover events, leading to higher probabilities of persistence that can set the stage for viral evolution to occur.

One Health decision-making is complex and in the case of SARS-CoV-2 in deer, our results suggest that indirect pathways warrant consideration when attempting to mitigate spillover and spread of SARS-CoV-2. Based on differences in spillover risk between direct and indirect transmission pathways, decision makers may consider a portfolio of management actions that mitigate multiple transmission pathways. In populations where wooded habitat conditions and deer density result in high rates of spread (*R*_0_ > 1), any form of spillover from any infectious host to deer, be it through direct or indirect transmission pathways, could result in a SARS-CoV-2 outbreak. In this case, decision makers may choose to focus on reducing both spillover and postintroduction spread, including reducing high deer densities, minimizing long duration of deer–deer proximity, and regulating use of attractants and supplemental feed [23, 45]. If a population's spread of SARS-CoV-2 is low (*R*_0_ < 1), decision makers may instead prioritize actions to reduce spillover risks, either from direct transmission from humans or from indirect transmission pathways. This prioritization is critical when a population's spread of SARS-CoV-2 could result in stuttering chains (0.5 ≤ *R*_0_ ≤ 1). Based on our work, direct transmission from humans poses the greatest spillover risk, and therefore, any reduction could alter rates of SARS-CoV-2 introduction into wild deer populations. However, management alternatives could be prioritized with focus on reducing spillover risk through indirect transmission pathways, particularly in areas with high human densities, and thus, higher risk of contaminated fomites.

This study demonstrates the potential risks posed by indirect transmission pathways and how they contribute to the maintenance of SARS-CoV-2 in wild deer populations. We report conditions where indirect transmission may be most important to consider across low-density deer ranges in the United States. While not sufficient as a standalone method to pinpoint specific locations and actions for risk mitigation, our findings suggest that decision makers could consider the roles of all viable transmission pathways, including those involving contaminated fomites when deciding whether and how to reduce the risk of SARS-CoV-2 spread in wild deer. These efforts could be aided by formal decision analysis methods, including structured decision making [46, 47], that may help in structuring decisions around localized contexts and that consider actions by multiple One Health sectors. Decision analysis methods can also inform where more precise parameter estimates would be helpful, including those involving indirect transmission pathways.

Our study may have broader implications for pathogen maintenance in a diversity of hosts and disease systems that include indirect transmission pathways. Wildlife species interact with their environment in complex ways that can facilitate indirect pathogen transmission, particularly through ingestion of concentrated food [48, 49]. Contamination of food surfaces and media from an individual host shedding pathogens can occur from settling respiratory droplets or biological fluid on contact [50]. Once contaminated, viral loads can persist on fomites for hours to days for some pathogens, including SARS-CoV-2; this prolonged viability external to hosts may lead to many opportunities for indirect transmission to occur [51]. Importantly, fomite transmission does not require both infectious and susceptible individuals to overlap in space and time [52]. Fomites present additional zoonotic transmission opportunities between humans and animals that may otherwise rarely overlap in proximity required for direct transmission [52].

Our SIRS approach and parameter set inherently assumed several characteristics of both simulated deer populations and the SARS-CoV-2 virus. Our inference that deer populations with low *R*_0_ are most sensitive to increases in FOI from direct and indirect transmission are based on naïve, randomly mixing populations exposed to a single variant of SARS-CoV-2. These conditions were appropriate for the purpose of this study as an initial exploration of disease dynamics; however, risk assessments for specific populations require careful consideration of these assumptions. Seroconversion has been detected in many wild deer populations across their range in the United States [14], potentially reducing the proportion of susceptible individuals in populations. Further, deer populations are likely not randomly mixed; social behavior and resource availability determine contact networks for a given deer population [53, 54]. Viral decay rate, infectivity, and the duration of infection and temporary immunity in deer likely vary across different SARS-CoV-2 variants, as documented in humans [55]. Modeling inferences and risk assessments could benefit from incorporating multiple variants and spatially explicit contact processes between individuals in simulations of populations previously exposed to SARS-CoV-2.

The indirect pathways altered outbreak dynamics in our simulations, yet more work remains in detecting and monitoring infectious SARS-CoV-2 virions on contaminated surfaces and in media. First, the efficacy for SARS-CoV-2 to infect a host when ingested during contact with fomites remains unknown. Our chosen dose–response function used parameters based on aerosolized transmission, which may overestimate the probability of infection from ingested virions [56]. While we reduced the dose received to account for the potential of reduced transmission efficiency (Equation 1 and Table S1), this knowledge gap warrants further study and may affect the risk posed by indirect transmission. Second, there have been limited studies demonstrating transmission risk via contaminated water sources, largely due to the challenges in isolating enveloped virions at low concentrations [57]. Secondary and tertiary wastewater treatments systems inactivate most SARS-CoV-2 virions, but the efficacy likely varies by treatment methodology [57, 58]. Further, untreated wastewater effluent is sometimes released into otherwise natural waterways during extreme flow events and mechanical failure at treatment facilities [29]. At the time of this study, there was no evidence of infectious virions in wastewater effluent. However, even a low risk of infection may cause spillover to wildlife populations drinking from these water sources, in addition to exposure to other contaminated fomites. Given our findings, further study of SARS-CoV-2 viability and dosages in and on contaminated fomites would be beneficial.

## 5. Conclusions

With the continued detection of SARS-CoV-2 in white-tailed deer and other wildlife species, the reservoir potential for SARS-CoV-2 in wildlife remains a concern. Despite a partial understanding of the modes of SARS-CoV-2 transmission, this study demonstrates that the rate of direct and indirect disease transmission may be important to pathogen circulation and maintenance. While the hazard posed by indirect transmission pathways are lower than direct transmission from humans, the cumulative hazard from both direct and indirect transmission pathways alter disease outbreaks beyond conditions predicted by epidemiological models that only consider direct transmission from humans. The apparent ease by which deer populations spread and sustain SARS-CoV-2 suggests that there may be multiple transmission pathways that contribute to the potential establishment of white-tailed deer as a reservoir species for a rapidly evolving zoonotic pathogen with profound human health implications.

## Data Availability

The R code underpinning this analysis is available through [57] and GitHub at https://github.com/erosenbl/Indirect_SARS.git. Data are publicly available in USGS ScienceBase https://doi.org/10.5066/P19KKRVV.

## Supporting Information

Supporting Information Table S1: Parameters used to estimate infection probability, force-of-infection, and various outbreak dynamics of SARS-CoV-2 in white-tailed deer (*Odocoileus virginianus*). Parameters were either fixed or drawn from a probability distribution as indicated in the description column. Table S2: Susceptible-infectious-recovered-susceptible (SIRS) ordinary differential equations (ODEs) used to project outbreaks of SARS-CoV-2 in simulated populations of wild white-tailed deer (*Odocoileus virginianus*) with and without the presence of facilities containing captive deer (wild-captive complex and wild, respectively). Figure S1: Sensitivities of outbreak metrics to categories of spillover (force-of-infection; FOI) and spread amongst white-tailed deer (*Odocoileus virginianus*; *R*_0_) in wild population exposed to both direct and indirect transmission pathways, with and without fence line transmission with captive deer (blue and green, respectively).
