# Supplementary material for: Fomites Could Determine Severity of SARS-CoV-2 Outbreaks in Low-Density White-Tailed Deer (Odocoileus virginianus) Populations
Source: Transbound Emerg Dis. 2025 Jun 4;2025:1352911. doi: 10.1155/tbed/1352911 (PMC12158583; doi:10.1155/tbed/1352911)
Supplement: Supporting Information — Table S1: Parameters used to estimate infection probability, force-of-infection, and various outbreak dynamics of SARS-CoV-2 in white-tailed deer (Odocoileus virginianus). Parameters were either fixed or drawn from a probability distribution as indicated in the description column. Table S2: Susceptible-infectious-recovered-susceptible (SIRS) ordinary differential equations (ODEs) used to project outbreaks of SARS-CoV-2 in simulated populations of wild white-tailed deer (Odocoileus virginianus) with and without the presence of facilities containing captive deer (wild-captive complex and wild, respectively). Figure S1: Sensitivities of outbreak metrics to categories of spillover (force-of-infection; FOI) and spread amongst white-tailed deer (Odocoileus virginianus; R0) in wild population exposed to both direct and indirect transmission pathways, with and without fence line transmission with captive deer (blue and green, respectively). [file 1352911.f1.docx]

**Supplemental Materials for**

Fomites could determine severity of SARS-CoV-2 outbreaks in low-density white-tailed deer (*Odocoileus virginianus*) populations

Elias G. Rosenblatt^1,*^, Jonathan D. Cook^2^, Graziella V. DiRenzo^3,4^, Evan H. Campbell Grant^5^, Michael C. Runge^2^, Brittany A. Mosher^1^

^1^Rubenstein School of Environment and Natural Resources, University of Vermont, Burlington, Vermont, United States of America

^2^Eastern Ecological Science Center, U.S. Geological Survey, Laurel, Maryland, United States of America

Massachusetts Cooperative Fish and Wildlife Research Unit, ^3^U. S. Geological Survey, University of Massachusetts, Amherst, Massachusetts, United States of America

^4^Department of Environmental Conservation, University of Massachusetts, Amherst, Massachusetts, United States of America

^5^S.O. Conte Anadromous Fish Research Laboratory, Eastern Ecological Science Center, U.S. Geological Survey, Turner’s Falls, Massachusetts, United States of America

*Corresponding Author: Elias G. Rosenblatt

**Email:**  [erosenbl@uvm.edu](mailto:erosenbl@uvm.edu)

Any use of trade, firm, or product names is for descriptive purposes only and does not imply endorsement by the U.S. Government.

**Table S1**: Parameters used to estimate infection probability, Force-of-Infection, and various outbreak dynamics of SARS-CoV-2 in white-tailed deer (*Odocoileus virginianus*). Parameters were either fixed or drawn from a probability distribution as indicated in the description column.

| Parameter | Description | Equation | Value | Source |
| --- | --- | --- | --- | --- |
|  |  |  |  |  |
| *p_ingested_* | Reduction in dose received via ingestion (uniform) | 1 | 0.1-0.9 | Assumed |
| *k* | Reciprocal of the probability of a single plaque-forming unit (PFU) causing infection | 1 | 420 | 1 |
| *PFU/ml_raw sewage_* | Concentration of PFU in contaminated sewage | 2 | 1.09 PFU/ml | 2; this study |
| *LR* | Log-reduction in PFU from sewage treatment (uniform) | 2 | 3-5 LR | 2 - 4 |
| *p_dilution_* | Dilution of treated wastewater in rivers (% of river water; logit-normal) | 3 | -0.7  (SD: 1.6) | 5 |
| *ρ_decay,water_* | Decay rate of SARS-CoV-2 in water | 3 | 1.1 days^-1^ | 6 |
| *t_decay, water_* | Residency time of SARS-CoV-2 in water before consumption (uniform) | 3 | 0-96 hours | Assumed |
| DWR | Daily water requirement of a white-tailed deer | 4 | 3.6 L | 7 |
| *gc_SARS-CoV-2_/ml_humansaliva_* | Concentration of SARS-CoV-2 genomic copies (gc) in human saliva (uniform) | 5 | 10^5.6^ gc/ml (SD: 10^1.2^) | 8 |
| *gc_SARS-CoV-2_/PFU_SARS-CoV-2_* | Conversion factor of genomic copies (gc) to PFU for SARS-CoV-2 | 5, 8 | 10^5.2^ gc/PFU | 9 |
| *r_apple core_* | Apple core radius (uniform) | 5 | 1.0 – 1.6 cm | This study |
| *h_apple core_* | Apple core height (uniform) | 5 | 5.4 – 7.6 cm | This study |
| *d_saliva_* | Depth of saliva surface in mouth | 5, 8 | 0.1 mm | 10 |
| *p_saliva transfer_* | Proportion of saliva in mouth transferred to apple (uniform) | 5, 8 | 0.1-0.9 | Assumed |
| *ρ_decay,fomite_* | Decay rate of SARS-CoV-2 on porous surface | 6, 9 | 4.8 days^-1^ | Modified from 11 |
| *t_decay, fomite_* | Residency time of SARS-CoV-2 on fomite before consumption (uniform) | 6, 9 | 0-96 hours | Assumed |
| *c* | Number of corn kernels contaminated (uniform) | 7 | 1-1000 kernels | Assumed |
| *p_contacted_* | Proportion of kernel surface area contaminated (uniform) | 7 | 0.01-0.5 | Assumed |
| *SA_k_* | Surface area of a corn kernel | 7 | 151.4 mm^2^ | 12 |
| *gc_SARS-CoV-2_/ml_deersaliva_* | Concentration of SARS-CoV-2 genomic copies (gc) in deer saliva (uniform) | 8 | 10^5.7^ gc/ml (SD: 10^5.3^) | 13 |
| *p_rivers_* | Proportion of surface water in rivers and streams (logit-normal) | 14 | -3.2  (SD: 1.4) | 14 |
| *p_contaminated_* | Proportion of rivers receiving wastewater effluent (logit-normal) | 14 | -1.0  (SD: 0.7) | 5 |
| *c_foodwaste_* | Consumption rate of food waste items (uniform) | - | 0-10 per day | Assumed |
| *c_feedpile_* | Consumption rate of feed pile servings (uniform) | - | 0-1 per day | Assumed |
| *α* | Immunity loss rate (log-normal) | Table S2 | 4.72  (SD = 0.63) | 13 |
| *γ* | Recovery rate | Table S2 | 1/6 days | 15 |
| β^Aero^_WW_ | Aerosol transmission risk between wild deer (log-normal) | Table S2 | -1.9  (SD = 1.3) | 13 |
| β^DC^_WW_ | Direct (Physical) contact transmission risk between wild deer (log-normal) | Table S2 | -6.5  (SD = 0.7) | 13 |
| β^Aero^_CW_ | Aerosol transmission risk between captive and wild deer across fencelines (log-normal) | Table S2 | -9.8  (SD = 1.4) | 13 |
| β^DC^_CW_ | Direct (Physical) contact transmission risk between captive and wild deer across fencelines (log-normal) | Table S2 | -14.4  (SD = 0.3) | 23 |
| β^Aero^_CC_ | Aerosol transmission risk between captive deer (log-normal) | Table S2 | 0.06  (SD = 1.6) | 13 |
| β^DC^_CC_ | Direct (Physical) contact transmission risk between captive deer (log-normal) | Table S2 | -5.5  (SD = 1.1) | 13 |
| β^Aero^_HW_ | Aerosol transmission risk between humans and wild deer (log-normal) | Table S2 | -10.3  (SD = 1.3) | 13 |
| β^Aero^_HC_ | Aerosol transmission risk between humans and captive deer (log-normal) | Tables S2 | -6.1  (SD = 1.6) | 13 |
| i_H_ | Human prevalence | Tables S2 | 5% | Assumed |

**Literature Cited**

1. Watanabe, T., Bartrand, T.A., Weir, M.H., Omura, T., Haas, C.N., 2010. Development of a dose‐response model for SARS coronavirus. Risk Analysis: An International Journal, 30(7), 1129-1138.
2. Zaneti, R.N., Girardi, V., Spilki, F.R., Mena, K., Westphalen, A.P.C., da Costa Colares, E.R., Pozzebon, A.G. and Etchepare, R.G., 2021. Quantitative microbial risk assessment of SARS-CoV-2 for workers in wastewater treatment plants. Science of the Total Environment, 754, p.142163.
3. Gundy, P.M., Gerba, C.P. and Pepper, I.L., 2009. Survival of coronaviruses in water and wastewater. Food and Environmental Virology, 1(1), pp.10-14.
4. Kumar, M., Alamin, M., Kuroda, K., Dhangar, K., Hata, A., Yamaguchi, H. and Honda, R., 2021. Potential discharge, attenuation and exposure risk of SARS-CoV-2 in natural water bodies receiving treated wastewater. npj Clean Water, 4(1), pp.1-11.
5. Ehalt Macedo, H., Lehner, B., Nicell, J., Grill, G., Li, J., Limtong, A. and Shakya, R., 2022. Distribution and characteristics of wastewater treatment plants within the global river network. *Earth System Science Data*, *14*(2), pp.559-577.
6. Bivins, A., Greaves, J., Fischer, R., Yinda, K.C., Ahmed, W., Kitajima, M., Munster, V.J. and Bibby, K., 2020. Persistence of SARS-CoV-2 in water and wastewater. Environmental Science & Technology Letters, 7(12), pp.937-942.
7. Lautier, J.K., Dailey, T.V. and Brown, R.D., 1988. Effect of water restriction on feed intake of white-tailed deer. The Journal of Wildlife Management, pp.602-606.
8. Buonanno, G., Stabile, L. and Morawska, L., 2020. Estimation of airborne viral emission: Quanta emission rate of SARS-CoV-2 for infection risk assessment. Environment International, 141, p.105794.
9. Lin, Y.C., Malott, R.J., Ward, L., Kiplagat, L., Pabbaraju, K., Gill, K., Berenger, B.M., Hu, J., Fonseca, K., Noyce, R.S. and Louie, T., 2022. Detection and quantification of infectious severe acute respiratory coronavirus-2 in diverse clinical and environmental samples. Scientific Reports, 12(1), p.5418.]
10. DiSabato-Mordarski, T. and Kleinberg, I., 1996. Measurement and comparison of the residual saliva on various oral mucosal and dentition surfaces in humans. Archives of oral biology, 41(7), pp.655-665.
11. Van Doremalen, N., Bushmaker, T., Morris, D.H., Holbrook, M.G., Gamble, A., Williamson, B.N., Tamin, A., Harcourt, J.L., Thornburg, N.J., Gerber, S.I. and Lloyd-Smith, J.O., 2020. Aerosol and surface stability of SARS-CoV-2 as compared with SARS-CoV-1. New England Journal of Medicine, 382(16), pp.1564-1567.
12. Karababa, E. and Coşkuner, Y., 2007. Moisture dependent physical properties of dry sweet corn kernels. International Journal of Food Properties, 10(3), pp.549-560.
13. Rosenblatt, E.G., Cook, J.D., DiRenzo, G.V., Grant, E.H., Arce, F., Pepin, K.M., Rudolph, F.J., Runge, M.C., Shriner, S., Walsh, D.P. and Mosher, B.A., 2024. Epidemiological modeling of SARS-CoV-2 in white-tailed deer (Odocoileus virginianus) reveals conditions for introduction and widespread transmission. *PLOS Compuational Biology*, *20*(7), e1012263.
14. ESRI, 2023. USA Detailed Water Bodies. February 6, 2023. https://hub.arcgis.com/datasets/esri::usa-detailed-water-bodies/about. (October 1, 2023).
15. Palmer MV, Martins M, Falkenberg S, Buckley A, Caserta LC, Mitchell PK, et al. Susceptibility of white-tailed deer (Odocoileus virginianus) to SARS-CoV-2. Journal of virology. 2021;95(11):e00083-21.

**Table S2**: Susceptible-Infectious-Recovered-Susceptible (SIRS) Ordinary Differential Equations (ODE) used to project outbreaks of SARS-CoV-2 in simulated populations of wild white-tailed deer (*Odocoileus virginianus*) with and without the presence of facilities containing captive deer (Wild-captive complex and Wild, respectively). These SIRS ODEs consider both direct transmission pathways between deer and between humans and deer, and indirect transmission via contaminated fomites. Wild-captive complex includes SIRS ODEs in both wild and captive populations, with the risk of fence line transmission via aerosols and direct contact ($\beta_{CW}^{Aero}$ and $\beta_{CW}^{DC}$, respectively). Notation includes superscripts to indicate the mode of transmission in wild deer (W), including aerosols (“Aero”); fluid exchanged through direct contact (“DC”); ingestion of water contaminated with wastewater effluent (“Water”); consumption of discarded food items contaminated by a human consumer (“Foodwaste”); and consumption of feed contaminated by infectious deer (“Feedpile”). Subscripts indicate the individuals in a particular transmission interaction: transmission between wild deer (WW); transmission between a human to wild deer (HW); fenceline transmission between captive and wild deer (CW) and indirect transmission to wild deer via contaminated surface (Indirect). Immunity loss rates (α), recovery rates (γ), and direct transmission rates ($\beta_{WW}^{Aero},\beta_{WW}^{DC},\beta_{CW}^{Aero},\beta_{CW}^{DC}, \beta_{HW}^{Aero}$) were estimated using parameters listed in Table S1.

| Context | Wild | Captive |
| --- | --- | --- |
| Wild | $\frac{ds_{W}}{dt}=\alpha r_{W}-s_{W}\left( \begin{aligned} \beta_{WW}^{Aero}i_{W}+\beta_{WW}^{DC}i_{W}+\beta_{HW}^{Aero}i_{H}+ \\ \beta_{Indirect}^{Water}i_{Water}+\beta_{Indirect}^{Foodwaste}i_{H} \\ +\beta_{Indirect}^{Feedpile}i_{W} \end{aligned} \right)$ | - |
|  | $\frac{di_{W}}{dt}=s_{W}\left( \begin{aligned} \beta_{WW}^{Aero}i_{W}+\beta_{WW}^{DC}i_{W}+\beta_{HW}^{Aero}i_{H}+\text{ } \\ \beta_{Indirect}^{Water}i_{Water}+\beta_{Indirect}^{Foodwaste}i_{H} \\ +\beta_{Indirect}^{Feedpile}i_{W} \end{aligned} \right)-\gamma i_{W}\text{ }$ | - |
|  | $\frac{dr_{W}}{dt}=\gamma i_{W}-\alpha r_{W}\text{ }$ | - |
| Wild-captive complex | $\frac{ds_{W}}{dt}=\alpha r_{W}-s_{W}\left( \begin{aligned} \beta_{WW}^{Aero}i_{W}+\beta_{WW}^{DC}i_{W}+\beta_{HW}^{Aero}i_{H}+ \\ \text{ }\beta_{CW}^{Aero}i_{C}+\beta_{CW}^{DC}i_{C}+ \\ \beta_{Indirect}^{Water}i_{Water}+\beta_{Indirect}^{Foodwaste}i_{H} \\ +\beta_{Indirect}^{Feedpile}i_{W} \end{aligned} \right)$ | $\frac{ds_{C}}{dt}=\alpha r_{C}-s_{C}\left( \begin{aligned} \beta_{CC}^{Aero}i_{C}+\beta_{CC}^{DC}i_{C}+ \\ \beta_{HC}^{Aero}i_{H}\text{ }\text{+}\text{ }\text{ }\beta_{CW}^{Aero}i_{W}+\beta_{CW}^{DC}i_{W} \end{aligned} \right)$ |
|  | $\frac{di_{W}}{dt}=s_{W}\left( \begin{aligned} \beta_{WW}^{Aero}i_{W}+\beta_{WW}^{DC}i_{W}+\beta_{HW}^{Aero}i_{H}+ \\ \text{ }\beta_{CW}^{Aero}i_{C}+\beta_{CW}^{DC}i_{C}+ \\ \beta_{Indirect}^{Water}i_{Water}+\beta_{Indirect}^{Foodwaste}i_{H} \\ +\beta_{Indirect}^{Feedpile}i_{W} \end{aligned} \right)-\gamma i_{W}\text{ }$ | $\frac{di_{C}}{dt}=s_{C}\left( \begin{aligned} \beta_{CC}^{Aero}i_{C}+\beta_{CC}^{DC}i_{C}+ \\ \beta_{HC}^{Aero}i_{H}\text{ }\text{+}\text{ }\text{ }\beta_{CW}^{Aero}i_{W}+\beta_{CW}^{DC}i_{W} \end{aligned} \right)- \gamma i_{C}\text{ }$ |
|  | $\frac{dr_{W}}{dt}=\gamma i_{W}-\alpha r_{W}\text{ }$ | $\frac{dr_{C}}{dt}=\gamma i_{C}-\alpha r_{C}\text{ }$ |


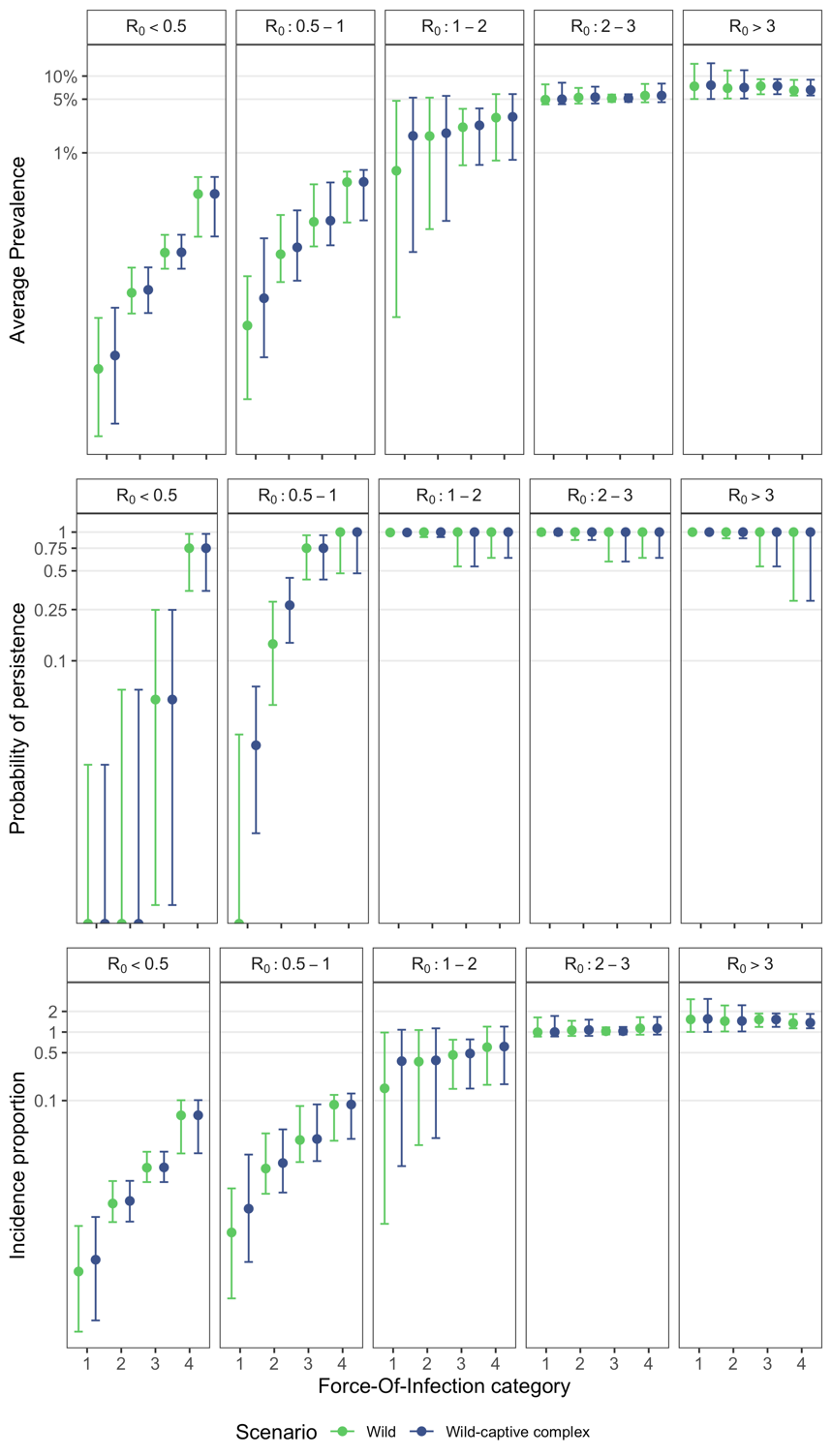


**Figure S1**: Sensitivities of outbreak metrics to categories of spillover (force-of-infection; FOI) and spread amongst white-tailed deer (*Odocoileus virginianus*; R_0_) in wild population exposed to both direct and indirect transmission pathways, with and without fence line transmission with captive deer (blue and green, respectively. We defined R_0_ categories of R_0_ < 0.5, 0.5 < R_0_ < 1, 1 < R_0_ < 2, 2 < R_0_ < 3, and 3 < R_0_, spanning conditions where spread is unlikely, spread is characterized by stuttering chains, spread is likely sustained, and spread is widespread, respectively. Force-of-infection categories include < 1.096x10^-5^ (1), 1.096x10^-5^ - 4.571x10^-5^ (2), 4.571x10^-5^ – 1.047X10^-4^ (3), and >1.047X10^-4^ (4). Points indicate median values from simulations that fall within each combination of FOI and R_0_ categories, with error bars indicating either 2.5 and 97.5 quantiles of simulation values (average prevalence and incidence proportion) or 95% confidence intervals (persistence probability).
